# Exploring links between common mental health problems, alcohol/substance use and perpetration of intimate partner violence: A rapid ethnographic assessment with men in urban Kenya

**DOI:** 10.1017/gmh.2017.25

**Published:** 2018-01-18

**Authors:** A. Schafer, P. Koyiet

**Affiliations:** 1 Technical Advisor Mental Health and Psychosocial Support, Humanitarian and Emergency Affairs, Global Rapid Response Team: Global Technical Team, World Vision International, Burwood East, Victoria, Australia; 2 National Coordinator for Gender, Disability, Mental Health and Psychosocial Support, World Vision Kenya, Nairobi, Kenya

**Keywords:** Aetiology, gender-based violence, intimate partner violence, Kenya, men, mental health

## Abstract

**Introduction:**

Kenya has some of the highest rates of gender-based violence (GBV) in the world, particularly intimate partner violence. World Vision completed a rapid ethnographic assessment to explore common problems faced by men and local perspectives about the links between men, mental health, alcohol use and GBV.

**Methods:**

Data from community free-listing surveys (*n* = 52), four focus group discussions and two key informant interviews formed the basis for thematic analysis and findings.

**Results:**

Lack of jobs, ‘idleness’ and finances were viewed as top priority concerns facing men; however, alcohol and substance use were equally prioritised. Family problems, crime and general psychosocial issues (e.g., high stress, low self-esteem) were also reported. Men withdrawing socially, changing behaviour and increasing alcohol consumption were described as signs that men were experiencing mental health challenges. The community observed alcohol use as the biggest cause of GBV, believing men resorted to drinking because of having ‘too much time’, marital conflict, psychosocial issues and access to alcohol. The findings theorise that a circular link between unemployment, alcohol and crime is likely contributing to familial, psychosocial and gender concerns, and that men's mental health support may assist to re-direct a trajectory for individuals at risk of perpetrating GBV.

**Conclusions:**

Data confirmed that GBV is a major concern in these Kenya communities and has direct links with alcohol use, which is subsequently linked to mental health and psychosocial problems. Attempting to disrupt progression to the perpetration of violence by men, via mental health care interventions, warrants further research.

## Introduction

Kenya has some of the highest rates of GBV in the world. According to the Kenya National Bureau of Statistics (KNBS & ICF Macro, 2010), more than 41% of Kenyan women experience sexual and/or physical violence by intimate partners in their lifetime, while in a 12-month period, 31% of women are living with active violence in their homes. This same Kenya report indicated that women are most commonly victims of GBV and men the perpetrators; although incidences of violence against men, perpetrated by women, are believed to be under-reported.

Gender-based violence (GBV) has no universal definition, but the World Health Organization (WHO) defines *interpersonal violence* as ‘*violence that occurs between family members, intimate partners, friends, acquaintances and strangers, and includes child maltreatment, youth violence, intimate partner violence (IPV), sexual violence and elder abuse*’ (WHO *et al.* 2014). GBV can mean different things according to culture, language and context; and it can incorporate subsets or types of violence and directions of victimisation/perpetration (Ellsberg & Heise, 2005). Nonetheless, GBV is a term generally accepted to feature violence against women, including IPV, on the basis that women are more likely to be victims of interpersonal violence perpetrated by men (ibid).

With support from World Vision in Canada and Australia, Grand Challenges Canada and working in partnership with WHO, University of New South Wales and Kenya's Ministry of Health, World Vision Kenya implemented a randomised control trial in 2016; which demonstrated effectiveness of PM+ among Kenya women affected by GBV (Bryant *et al.* 2017). During this work, consultations with the community heard them repeatedly seek to primarily prevent, and secondarily reduce violence against women in addition to responding to those affected. Women described men's alcohol consumption, drug use, lack of employment and poverty-related stressors to be substantial contributors to GBV, and it was suggested that GBV prevention and reduction may be achieved by addressing common mental health problems among men in the community (Schafer *et al.* 2013; Schafer, 2014). This rationale has long-held support in GBV literature, particularly in relation to reducing harmful alcohol consumption (Jewkes, 2002).

### Men, mental health, alcohol/substance use and reducing GBV

It is accepted that prevention of GBV needs to employ an ecological approach targeting, in parallel, risk factors at all levels – societal, communal, family/relational and individuals (WHO *et al.* 2014; IASC, 2015; UNWomen *et al.* 2015). Yet current evidence on the effectiveness of interventions to prevent and reduce GBV currently shows mixed results (Fulu *et al.* 2014; UNWomen *et al.* 2015), particularly in low- and middle-income countries (LMIC). Fulu *et al.*’s evidence review of the literature on violence prevention initiatives found that certain interventions could be grouped as having effective, promising, conflicting and ineffective outcomes. Effective outcomes included community-based transformative approaches (e.g. microfinance and empowerment for women) and men-focused programmes (e.g. group education, outreach). However, Fulu *et al.* did not extend the review to examine targeted mental health care for men experiencing mental health problems as a potential GBV reduction strategy, even though high-income countries have shown evidence in line with this idea (WHO *et al.* 2014; UNWomen *et al.* 2015).

The UN Women's framework for action (2015) to prevent violence against women highlights risk factors for perpetration, including mental health problems and various social factors known to be risks for mental health difficulties. The framework cites depression, low life satisfaction, individual experiences or witness of violence, alcohol use, marital discord and low social connectedness as potential contributors to the perpetration of violence. The framework refers to growing evidence that mental health treatment for both perpetrators and victims of violence may not only help prevent violence, but also stem the flow of revictimisation: ‘*Poor mental health is a risk for both victimization and perpetration, suggesting the importance of integrating strategies to prevent violence against women into programmes to prevent and respond to poor mental health*’ (p. 41). This position to integrate GBV response and prevention, in line with mental health services, echoed the previously published WHO Global Status Report on Violence Prevention (2014).

In Kenya, one of the most challenging mental health concerns, particularly among men, is heavy alcohol consumption and abuse (KNBS & ICF Macro, 2010). Alcohol abuse and mental health problems often go hand in hand (WHO, 2004; WHO *et al.* 2014; Grant *et al.* 2015), forming a negative cycle. Individuals with common mental health problems use alcohol as a coping strategy, which worsens mental health, social and familial problems. This is a cycle also shown to exacerbate violence (Fulu *et al.* 2013) and poverty (WHO, 2010*a*).

In Kenya's health surveys conducted in 2009 (KNBS & ICF Macro, 2010), findings confirmed that women whose husbands were often drunk were twice as likely to experience emotional, physical or sexual violence (79%) compared with women whose spouses did not consume alcohol (39%). A logical step might be to solely decrease individuals’ consumption and harmful use of alcohol. However, global mental health research stipulates that singular mental health interventions for singular mental disorders/problems are not sustainable, scalable or effective to treat the myriad common mental problems in LMIC (IASC, 2007; WHO, 2010*b*). This is especially true since alcohol and substance use disorders are so commonly co-morbid with other mental disorders (Grant *et al.* 2015). Therefore, WHOs PM+ intervention (WHO, 2016) may be suitable to the Kenya context. It is evidence-based in LMIC (Rahman *et al.* 2016; Bryant *et al.* 2017); it manages multiple common mental health concerns without specific diagnosis; it is brief (five sessions) and has a practical behavioural/problem-solving focus. PM+ also shows promise for scalability with community volunteers shown to deliver the intervention effectively (Dawson *et al.* 2015).

### Goals of the ethnographic assessment

Prior to World Vision Kenya rolling out a group version of PM+ for men with common mental health problems in a definitive feasibility study, it was necessary to initially explore various factors: issues facing men in the intervention communities; presentations of mental health problems; local perspectives about men, and their mental health, alcohol use and GBV. To achieve this, an exploratory rapid ethnographic study was undertaken aiming to examine:

1. Common problems faced by men and how people prioritise those problems;
2. Descriptions of how men with common mental health problems present;
3. Understanding of local perspectives about GBV (IPV), as they relate to men, and explore possible causes, justifications, belief and attitudes;
4. Understanding local beliefs about alcohol and substance use;
5. Ways men seek support for mental and emotional issues within their community and common coping strategies; and
6. Links between mental health, alcohol and substance use and violence against women in these target communities.

## Method

### Rapid ethnographic assessment

Ethnography is a descriptive study of cultures, groups, customs and habits. It is usually based on open-ended interviews and grounded in learning from people rather than studying or analysing their behaviour against norms (Beebe, 2001). Traditional ethnography is a long qualitative process, but rapid ethnography is now viewed as standard practice for cross-cultural health and social sciences where time-sensitive information is required and limited resources prevent more extensive research (Bolton, 2001; WHO & UNHCR, 2012). A key methodological consideration for rapid ethnography is applying triangulated data, which combine qualitative and non-powered quantitative results. Ethnography has been effectively used in Africa as a way to adapt mental health care interventions to local context (e.g. Interpersonal Psychotherapy for Groups; Bolton *et al.* 2003) and also formed part of the pilot approaches to the PM+ pilot and randomised control trail in Kenya (Schafer *et al.* 2013; Schafer, 2014; Dawson *et al.* 2015).

This study followed the rapid ethnographic process from Tool 10 of the *WHO & UNHCR (2012) Assessing Mental Health and Psychosocial Needs and Resources: Toolkit for Major Humanitarian Settings*. Adaptations ensured that the community free-listing survey was specific to the objectives of this particular assessment. Annex A provides a copy of the community survey tool used. Further qualitative information was collected using focus group discussions (FGDs) and key informant interviews (KIIs), both of which were loosely guided by questions about GBV, men and mental/emotional problems affecting them. Guidance questions for FGDs and KIIs are outlined in Annex B.

### Participants

To undertake the community free-listing survey, six male and six female Kenyan enumerators were employed; they all had prior experience in research and data collection. Enumerators received 3 days participatory training (including role-plays) on the current research goals, qualitative interviewer skills, ethics, appropriate use of probing questions and accurate note-taking and reporting. They were additionally briefed on the convenience sampling approach where they were eventually transported to the centre of the town and asked to seek informed consent from random individuals to complete the survey – roughly seeking information from people in different parts of the town and different age groups. Mostly, male interviewers spoke with male respondents (with the female note-taking), while female interviewers spoke with female respondents. They were asked to interview approximately 10 individuals per data collector ‘pair’, comprising a 60% male/40% female split reflecting the emphasis of the exploratory study on men's mental health and beliefs about GBV. Free-listing surveys were completed using multiple languages including English, Swahili and Kikuyu. Information was recorded in English. Efforts to mitigate translation errors were made with both interviewers instructed to compare notes, information and language translation after each interview; attempting to ensure data recorded in English were as accurate and representative of respondent views as possible.

Based on the World Vision Kenya's long-standing agreement with the local government, administration and communities under study, independent ethics approval was not deemed necessary. Nevertheless, all survey participants were read information about the study and their voluntary informed consent was sought. Further, all female enumerators hired for this work had previously received training in Psychological First Aid to be able to support respondents who may have expressed or shown signs of distress.

Free-listing data comprised 52 respondents: 13 men and eight women from Waithaka and 20 men and 11 women from Muitini (total of 33 men and 19 women). Average age of respondents was 33.08 years (s.d. = 11.70 years). FGDs were undertaken with participants identified by the authors [as recommended by the *WHO & UNHCR (2012) Assessing Mental Health and Psychosocial Needs and Resources: Toolkit for Major Humanitarian Settings*], as being important community members to discuss these topics with. FGD participants were selected by local World Vision Kenya staff who have worked among these communities for more than 14 years.

Four FGDs were undertaken with: (a) men from Waithaka; (b) men from Muitini; (c) female community health workers previously trained and experienced in implementing PM+ with women; and (d) male community leaders, which included four community elders, a community chief (from one of the two community areas), a local police officer, a pastor and a community-based organisation leader. Two KIIs were also held with a male health nurse at a primary health care facility and another community chief, since these were identified in the FGDs as important community roles commonly required to manage issues relating to GBV and men's mental health. All FGDs and KIIs were led by the author (A.S.) and held in English. A trained enumerator (male) was also present during all interviews so that some local concepts or terms could be adequately translated and explained. The author and enumerator both took handwritten notes during FGDs and KIIs, comparing key points and information at the conclusion of each process.

### Data treatment

Results are presented according to the areas of exploration of the ethnography, drawing mostly from findings of the free-listing interviews, but supported and triangulated with corroborating data from the FGDs and KIIs. The results represent a summary of information obtained, and are organised according to the key themes that emerged during analysis.

In the free-listing exercise, after respondents identified problems, they were asked to rank them in order of priority, according to what they viewed as the most, second most and third most important issues facing men. For analysis, themes mentioned as most important were scored three points each; second most important, two points each; and third most important, one point each; which allowed for a calculation of a total score to represent an overall perspective of which issues were the strongest priorities for communities.

Qualitative information and thematic analyses were led by the author (A.S.) with support from six of the 12 data collectors during a 2-day workshop. Responses to the surveys were coded and thematic summaries applied, expanded, reviewed, and named, following the six phases of the thematic analysis outlined by Braun & Clarke (2006). Transcripts of the FGDs and KIIs were discussed and examined, and they provided support to information extracted during the free-listing analyses. Throughout the analysis, some interpretive meaning of responses was applied, using an Interpretive Phenomenological Approach (IPA) recommended by Smith & Osborn (2008). IPA aims to unravel perceptions or accounts according to their underlying meaning, or sentiment, as opposed to concentrating on the literal terms or information provided. Combining analytic approaches for qualitative and/or ethnographic data is viewed as good practice to enhance the credibility of results (Magg-Rapport, 2000; Leech & Onwuegbuzie, 2008). Finally, results were organised to correspond with the six goals of the ethnographic study and are presented accordingly.

## Results

### Common problems faced by men and how communities prioritised those problems

The free-listing exercise explored the kinds of problems men in these communities faced. Seven themes emerged. Issues related to lack of jobs, subsequent ‘idleness’ and lack of finances were the top priority; however, this was almost matched by the number of respondents who reported alcohol and substance use/abuse being a major problem faced by men in their communities. Family or marital problems and crime were next to be listed as major concerns. Generic psychosocial issues referred to direct statements about individuals feeling high stress or low self-esteem. It was notable that the single word ‘stress’ was frequently mentioned across these seven themes. A summary of the most common problems faced by men, according to the thematic content in this survey, were unemployment, alcohol and substance use, family problems, crime, general psychosocial issues, gender issues and other non-specific issues.

When ranking the prioritisation of the problems identified (as first, second and third highest priorities), analyses showed a clear pattern that unemployment, and alcohol and substance use were the standout priorities in this order. As a ‘top three’ list of priorities, these were identified well above any other concerns. Descriptions of the seven themes communities identified as facing men and their ranked total scores are provided in Table 1.

**Table 1.** Summary themes and prioritisation for what communities viewed to be the most important problems facing men in Waithaka and Muitini

Themes, theme descriptions and number of responses	Total ranked score	Qualitative excerpts
**‘Unemployment’**, financial problems and ‘idleness’ (as a consequence of unemployment): *n* = 65	123	Due to idleness, and lack of opportunities. There is a high level of unemployment Money is hard to come by and life becomes hard A man without money is considered hopeless since he cannot provide for himself Because of not having anything to do, men start thinking of taking alcohol, having affairs with other women and neglecting their families
**‘Alcohol and substance use’** and abuse: *n* = 63	113	Most men are stressed so they indulge in alcohol to escape Men become unproductive. They feel they are in their comfort zone when taking drink and it gets him away from responsibility Because they have neglected the families and women are the ones who take care of the families. It's [alcohol] readily available and cheap Many young men are hooked to drugs and many are unable to function well in society
**‘Family problems’**, including separation, unmarried men, familial conflict, domestic violence: *n* = 23	17	Lack of money leading to stress which is channelled to the family Husband beats wife when asked about money. Man doesn't go to work to punish the woman Marital problems make men to behave weirdly as a way of showing their anger Quarrelling because of lack of money and financial constraints
**‘Crime’** and police harassment: *n* = 20	12	Petty crimes to satisfy their craving for alcohol and drugs Harassment by police and city council in jobs Men steal money from other people and end up drinking alcohol with the money
General **‘Psychosocial issues’**, such as stress, low esteem, peer pressure: *n* = 9	9	They [men] do not have avenues to air their problems or talk about their challenges They [men] have lost hope in life and they just live a day at a time A stressed person gets to drinking as a way of relieving stress
**‘Gender issues’**, such as men feeling disempowered, perceived preferential treatment of women, gender inequality: *n* = 6	6	Women are being sought for jobs more than men Men cannot access loans as easily as women do. Women are given priority first over men Men are not given much attention, like girls. And therefore, men end up doing things because of pressure from society to provide basic needs
**‘Other’**, with single mentions for issues including low education, local environment, tribalism and three mentions of diseases including HIV/AIDS (*n* = 1) and diabetes (*n* = 2): *n* = 7	*	The environment [in Waithaka] is not good to bring up a child Increase in diseases, such as diabetes, cancer, blood pressure Tribalism; leads to men not being secure in a job because of his tribe No quality education and no facilities in school. Most youth depends on their parents and don't want to work hard

*‘Other’ issues were not ranked in order because it represented a collection of ‘other issues’, most of which were unique and could not be collectively ranked.

### Presentation of men with mental health problems

When asking how men might be identified as experiencing mental health or emotional problems, nine themes were described. Social withdrawal, behaviour change, alcohol and drug use, and noticeable behaviour changes were the most prominent ways people observed a men to be struggling. Themes were:

1. **Social withdrawal**: *n* = 20
2. **Behaviour change**, including becoming violent, aggressive or turning to crime: *n* = 14
3. **Alcohol and drug use**: *n* = 13
4. **Mood changes**, such as showing low or changing moods, sadness, stress, depression or silence: *n* = 11
5. **Poor concentration**, including absent-mindedness, confused thinking or inattentiveness: *n* = 8
6. **Poor hygiene**, including poor appearance and presenting oneself as ‘unkempt’: *n* = 8
7. **Neglect of family**, responsibilities and work (including being idle): *n* = 8
8. **Being open** and talking to others or sharing their problems: *n* = 8
9. **Poor physical health**: *n* = 7

### Perspectives about GBV (including IPV) as it relates to men, including possible causes, justifications, beliefs and attitudes

The free-listing survey sought specifically to understand ideas about why men perpetrate IPV, broadly referred to locally as GBV. In response to this question, six key ideas were put forward, with alcohol, marital problems and the behaviour or actions of women perceived to be the main causes.

1. **Alcohol and drug use** were seen to be directly linked to violence, including IPV: *n* = 20. For example: ‘The men abuse alcohol hence it leads to violence’; ‘The men get minimum wages and to avoid being questioned by the wife – he goes home drunk and beats up the wife’.
2. **Martial conflicts**, problems, infidelity, poor communication and lack of dialogue were often referred to as causes for IPV: *n* = 15. For example: ‘Men perpetrate violence because of domestic affairs by their wives’; ‘No communication and understanding between the men and women’.
3. **Financial** and related employment challenges were also viewed as a cause, particularly with its perceived link to alcohol and drug abuse: *n* = 12. For example: ‘Because of lack of job opportunities they resort to abusing women because of the pressure to provide’; ‘Rape cases are on the increase because most men are idle [jobless], which results in bestiality, alcohol and substance abuse, which are a major reason for wife-beating’.
4. **Psychosocial issues**, such as stress, men feeling a need to prove themselves or escape from family responsibilities were suggested as possible causes: *n* = 11. For example: ‘Anger among men’; ‘Men have ego issues and therefore when they abuse women, they want to prove they are men and have power’; ‘Stress and not opening up to anyone, thus projecting anger to wife’.
5. **Women's actions** were commonly cited as being the reasons for inciting violence against them, including perceived inappropriate dress, not providing conjugal rights to their husbands, attitudes of women towards men and women's unfaithfulness in marriage: *n* = 10. For example: ‘It is done because women have bad behaviours. Taking alcohol, their dress code is not right’; ‘Disrespect by women [towards men] has led to gender violence’; ‘It happens because of [men] being ignored by women [sexually], hence rape’.
6. **Cultural beliefs** and attitudes about men's ‘traditional’ relationship with women were further considered a contributor for perpetrating violence against women: *n* = 8. For example: ‘They [men] think it's their right’; ‘Culture and beliefs, in that they [men] believe that a wife should be battered once in a while’; ‘Some women are not submissive so the men beat them’.

There was one statement in this specific survey section about men being victims of violence perpetrated by women: ‘*It is because they* [*men*] *come home with no food and thus why they* [*men*] *are beaten*’. However, the theme of men being victims of violence perpetrated by women was raised in various ways throughout free listing, the FGDs and KIIs. In particular, it was noted that when a man is a victim of violence, it is especially difficult for him to reach out for support since it would be viewed culturally as shameful to experience such problems, as described in these statements:

- *Because of financial constraints, they [the men] are abused by their wives*;
- *Some men are victims of GBV and end up taking alcohol and abusing drugs*;
- *Some men are beaten by their wives and men fear reporting such cases to policemen due to shame*.

### Beliefs about alcohol and substance use

It was anticipated that alcohol and substance use would be viewed as common problems and possible causes for GBV in these communities. Therefore, the free-listing survey asked directly about this topic. The most common reported causes indicated for excessive alcohol consumption was ‘too much time’, closely followed by psychosocial issues and the proximity and cheap availability of alcohol. Cultural beliefs about men and alcohol consumption being socially sanctioned were also featured in participants’ responses.

### Ways men seek support for mental and emotional issues

Participants were asked what men might do to manage their problems. Responses were themed according to three broad categories – positive coping strategies, unhelpful coping strategies and doing nothing at all. Within positive and unhelpful coping strategies, some sub-categories were identified and shown in Table 2.

**Table 2.** Summary of coping strategies communities identified as being commonly used by men seeking support for mental health and/or emotional problems

Positive coping approaches	Talking to family or friends (*n* = 19)	Sharing problems with family or friends Engaging in conversations with friends Participating in family activities together Seeking advice
	Approaching the chief, community elders or local parliamentary members for support (*n* = 3)	Village elders, to MPs (Members of Parliament) They go to the chief to try and solve problems
	Prayer and church attendance (*n* = 5)	Men to go church for spiritual nourishment Praying to God to help you
	Focusing on work (*n* = 13)	To sacrifice and be willing to take part in any job, even if it's an odd job Adopting a lifestyle that can be sustained by your income Keeping themselves busy and also setting up businesses
	Moving away from unhelpful influences, such as reducing alcohol intake (*n* = 5)	Regulation of time for drinking/taking alcohol Refusing to use alcohol or putting down some rules on alcohol use
Unhelpful coping strategies	Turning to alcohol and drugs (*n* = 11)	Alcohol indulgence to forget their problems Drinking with friends
	Resorting to violence and crime (*n* = 6)	They get violent, especially at home towards children or the wife Men become defensive by fighting them [other men]. They go into crime
	Suicide (*n* = 3)	Some commit suicide
Doing nothing	Ignoring problems, not taking any action or not having avenues to seek help (*n* = 6)	Most men do not open up to talk about their problems, but instead, they bottle up everything inside. They do not seek help They stay with them [their problems]. They do not accept they have a problem

At the conclusion of the free-listing survey, participants were asked if there was anything else important for the researchers to know about men in the communities. At this point, 19 out of 41 individuals who made additional comments indicated that support for men was needed in their community. Kenyan enumerators, FGD and KII participants indicated that men felt strongly about this need on the basis of so many years of focus on women's issues or ‘the girl child’. Recommendations made for supporting men ranged from assisting men to be educated more about social issues, alcohol and violence, to holding seminars and workshops and providing more public facilities for sports and recreation. Specifically, many also mentioned the need to offer counselling-type support services for men. For example:

- *We need men to form support groups to share their challenges and the way forward*;
- *Organising forums for men to exchange ideas. Peer education and guidance and counselling for men*;
- *We need to move from our cocoon that a man should not cry … It is important for us to move away from that culture and understand that it is OK for a man to cry and to share his problems*.

### Links between mental health, alcohol and substance use and violence against women

**Fig. 1.**
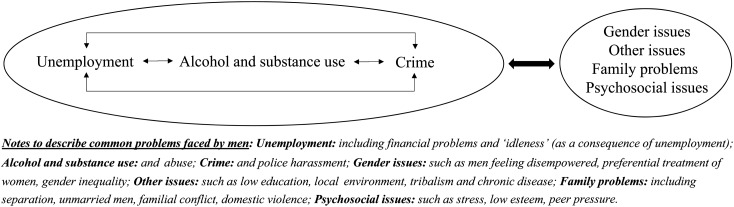
Circular links between common problems faced by men in Muitini and Waithaka, Kenya.

The data suggested two clear areas of common mental health problems: practical and behavioural problems, such as unemployment, alcohol use and crime; and broader issues such as gender, other concerns and psychosocial or familial issues. The practical and behavioural challenges show a circular link: unemployment leads to alcohol and substance use, which leads to crime; while the combination of crime and/or alcohol/substance use was reported to contribute to unemployment. These combined issues appear to be exacerbating psychosocial issues, familial problems, gender perceptions in the community and other challenges; and *vice-versa*. To restate these linked themes, Fig. 1 depicts a flow diagram summarising how these constructs were reported in the data.

**Fig. 2.**
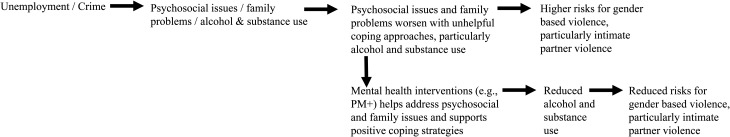
Theoretical progression of common problems faced by men in Muitini and Waithaka, Kenya, leading to risk for perpetration of GBV, and potential for reducing risk by supporting men's mental health.

The challenge with these circular links is that they do not clearly identify where mental health and psychosocial support – particularly for men – might contribute to reducing GBV. Therefore, to break down these cycles, Fig. 2 attempts to show a theoretical link that is more linear in nature, but true to the patterns communities identified. The progression to GBV, specifically IPV, could be described to show that unemployment and crime lead to psychosocial issues, family problems and alcohol use; most likely viewed as a consequence of stress, anxiety or difficulty coping with the problems of unemployment and the financial constraints this places on families. Unhelpful coping strategies, including alcohol and substance use, worsen psychosocial and familial problems and in turn, heighten the risks of IPV in the household, and overall GBV in the community. In theory, by intervening for men experiencing common mental health issues and wider psychosocial problems, use of alcohol as a coping strategy could be reduced and thus IPV might, at an individual level, also reduce or be prevented.

## Conclusions

Highlights of results showed that unemployment and its related consequences – particularly financial problems and ‘idleness’ as well as alcohol and substance use – were perceived by the Waithaka and Muitini communities to be the most common problems faced by men. Additional problems included family difficulties, psychosocial and gender issues. Unemployment and alcohol/substance use were, respectively, viewed as being the first and second most important issues facing men, with family problems identified as the third most important. Men with mental and emotional problems were known in their communities because they were socially withdrawn, show behavioural changes, increase alcohol consumption, experience mood changes and various other signs indicating distress.

When communities were asked why men were perpetrating violence against women, alcohol and marital conflict were viewed as the biggest causes, with finances (and related lack of employment) only viewed as the third most cited cause; although this was commonly highlighted as the basis for the marital conflict. It appears men were turning to alcohol as a result of having too much time, as a consequence of unemployment (or underemployment), psychosocial problems and because alcohol was said to be widely and cheaply available in the communities. This offers the first indication that supporting men's mental health and psychosocial concerns could be important to reducing alcohol use and potentially help to prevent or reduce violence in these communities; especially since men were commonly conveyed as using alcohol for coping with adversity, despite numerous positive coping strategies being available. Further, there was a common, unexpected theme that many believed men with mental health problems suffered in silence and had few, if any, accessible supports. There was expressed frustration among men in the community about a perceived inequality with prior programmes focused more strongly on women and the ‘girl child’ and meeting their practical and mental health needs, leaving men behind. PM+ may help bridge a gap in the community to offer such emotional and mental health support to men as well as women.

The data also implied an alignment with the possibility that men may do well with a group version of PM+ (WHO, 2016). This conclusion is based on the PM+ focus on stress management, practical problem-solving and strengthening positive social supports as a helpful coping strategy (Dawson *et al.* 2015).

This rapid ethnographic assessment was exploratory in nature and based on the need to confirm if the intended intervention, Group PM+, might be relevant and helpful for Kenyan men in a definitive feasibility study. As such, a number of limitations to these findings must be considered. Community participants were not randomly or systematically selected and data collectors may have shown bias towards interviewing those more easily accessible. Further prospective biases to the interpretation of findings are another limitation given multiple phases where such biases or misinterpretations could have occurred; for instance, during data recording (from local languages to English), the male/female data collectors who were always present during interviews with male/female respondents and the leadership of the data treatment by the primary author (A.S.), with latent subjectivity to the themes allocated possible. Nonetheless, efforts were made to kerb these limitations through the inclusion of local enumerators, data collection undertaken in pairs and multiple sources of information used to generate key findings. The author also engaged the local data collection team to confirm thematic results reported. Furthermore, themes identified matched those formerly detected in previous ethnographic assessments (Schafer, 2014) and literature that shows irrefutable correlations between alcohol and substance use and GBV (Jewkes, 2002; KNBS & ICF Macro, 2010; Fulu *et al.* 2013; WHO *et al.* 2014); and aspects of what the UNWomen's *et al.* (2015) framework ascertained as promising practices for reducing GBV in LMIC.

This study confirmed that GBV/IPV is a major concern in these two urban communities in Kenya and it appears to be strongly linked with alcohol use or abuse. The data maintained that men may be inclined towards perpetration of violence against women given stressors such as unemployment, excessive alcohol and substance use, family difficulties and marital conflict as well as other psychosocial, cultural and gender issues. The data suggested a cyclic link between the impacts and causes of violence. It seemed that men who experienced stress tended to consume more alcohol; and men who consumed more alcohol might be more likely to perpetrate GBV. Therefore, attempting to disrupt this cyclic progression, by supporting men with high stress or other common mental health problems, could help reduce the trajectory to GBV perpetration; although this pattern requires further study, possibly with attention to quantitative information that tracks these causal pathways.

Another important issue to examine in the prospective feasibility study to proceed this rapid ethnographic assessment will be in relation to how well Group PM+ supports men with more serious substance and alcohol use/abuse problems. PM+ is an intervention designed for common mental health problems, but not specifically for chronic or longer-term substance/alcohol use. It will be essential to consider possible cut-offs for individuals with severe substance/alcohol use disorders and their participation in the Group PM+ programme, and to carefully monitor impacts Group PM+ has on consumption in the immediate and long term.

It is critical to note that while addressing men's mental health needs in communities at an individual level could help reduce GBV, additional social and cultural changes will be important to achieve wider change. This was commonly mentioned throughout the ethnographic results. Notably, the need to work towards attitudinal changes to cultural beliefs that men have rights to beat their wives, advocating for reduced availability and affordability of alcohol and expanding employment opportunities would likely go a long way to curtailing GBV in these communities. Although such interventions are beyond the scope of the current World Vision Kenya project, these findings echo those of others and emphasise the need for ecological, holistic approaches to GBV prevention (Fulu *et al.* 2014; WHO *et al.* 2014; IASC, 2015; UNWomen *et al.* 2015).

In summary, data have substantiated that a mental health intervention for men, such as Group PM+, might yield benefits to assist men to better manage common mental health and psychosocial problems; and potentially contribute to individual reductions and/or prevention of IPV via lessened alcohol and substance use. The intent of the wider project aim and objectives is aligned with the problems being faced by men in Waithaka and Muitini and findings from this study suggest further research, such as a feasibility study, is warranted.

## Supplementary material

For supplementary material accompanying this paper visit http://dx.doi.org/10.1017/gmh.2017.25. click here to view supplementary material
