# Supplementary material for: Exploring links between common mental health problems, alcohol/substance use and perpetration of intimate partner violence: A rapid ethnographic assessment with men in urban Kenya
Source: Glob Ment Health (Camb). 2018 Jan 18;5:e3. doi: 10.1017/gmh.2017.25 (PMC5797935; doi:10.1017/gmh.2017.25)
Supplement: Supplementary file 1 [file gmhsup.zip › S2054425117000255sup001.docx]

Annex A: Community Free Listing Survey

This survey was adapted from Tool 10 in: World Health Organization & United Nations High Commissioner for Refugees. *Assessing Mental Health and Psychosocial Needs and Resources: Toolkit for Major Humanitarian Settings*. Geneva: WHO, 2012.

Some information collected in this survey were not reported as part of the ethnographic study (e.g., questions 11, 12, 13, 16) because they were used specifically to adapt measures planned for the definitive feasibility study. Therefore, not all findings form the survey have been reported as part of the ethnographic analyses. Layout of the survey was edited for field-use.

**
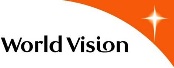
**

**Community Free-Listing Survey**

**Informed consent:** Hello, my name is _________________ and I work for World Vision. We have been working in Muitini and Waithaka to try and address common mental health problems affecting men and women, with a particular focus on sexual and gender based violence. We have worked a lot with women in the past, but our new project aims to work with men. Today, we are trying to learn more about the kinds of problems men in this community face so we can decide how we can best offer them support. We cannot promise to give you support in exchange for this interview. We are here only to ask questions and learn from your experiences. You are free to take part or not. The interview will take about 15 minutes. If you do choose to be interviewed, I can assure you that your information will remain anonymous so no-one will know what you have told us. We cannot give you anything for taking part, be we would greatly value your time and responses. Do you have any questions? **Do you agree to be interviewed? YES / NO**

| Demographic Data: | | INTERVIEWER NAMES: |
| --- | --- | --- |
| Location: | MUITINI WAITHAKA | Interviewer: |
| Gender: | MALE FEMALE | Note-taker: |
| Age: |  |  |

| Q1: What kind of problems do men in this community face? Please tell us as many problems that you can think of. *Probe: Can you think of other problems men face?* | Q2: Can you describe for me each of these problems you listed? | FOR INTERVIEWERS ONLY: Tick if this problem is related to mental health problems |
| --- | --- | --- |
| *Problems* | *Description* | *Is this problem related to mental health issues?* |
|  |  |  |

| Q3: You mentioned a number of problems, including [*list mental health problems*]. Of these problems, which is the MOST important problem facing men? | Q4: Can you tell me why you see this as being the most important problem facing men? |
| --- | --- |
|  |  |
| Q5: Of these problems, which do you think is the second most important problem facing men? | Q6: Why? |
|  |  |
| Q7: Of these problems, which do you think is the third most important problem facing men? | Q8: Why? |
|  |  |

Q9: We are told that sexual and gender based violence is common in this community. Can you share with us your thoughts about why men may perpetrate this kind of violence?

Q10: We are told that alcohol use is common in this community. Can you share with us your thoughts about alcohol use amongst men?

| Q11: What are the activities men do frequently to care for themselves? | Q12: What are the activities men do frequently to care for their families? | Q13: What are the activities men do frequently as part of their community? |
| --- | --- | --- |
|  |  |  |

Q14: Sometimes, it can be difficult for men to cope with everyday problems and perform their usual tasks. How would other people know if a man was struggling with their problems?

Q15: What kinds of things do men do to deal with such problems? For example, things they might do themselves, with their families or with others?

Q16: If a man in this community was experiencing mental or emotional problems where would they seek help? *Probe if not mentioned: Would this include seeking help for problematic alcohol use?*

Q17: Is there anything else about men in this community that you would like to share with us, or think is important for us to know?

Other Notes:
