# Supplementary material for: Exploring links between common mental health problems, alcohol/substance use and perpetration of intimate partner violence: A rapid ethnographic assessment with men in urban Kenya
Source: Glob Ment Health (Camb). 2018 Jan 18;5:e3. doi: 10.1017/gmh.2017.25 (PMC5797935; doi:10.1017/gmh.2017.25)
Supplement: Supplementary file 1 [file gmhsup.zip › S2054425117000255sup002.docx]

Annex B – Guide for key informant interviews and community focus group discussions

The focus group discussions and key informant interviews were informal discussions, and deliberately open-ended, conducted with minimal guidance. The following format was used as a general guide, but discussions flowed from information raised by participants.

*Introductory remarks:*

- Introduction to the previous work World Vision Kenya did with women affected by gender based violence (GBV) and the intention of now working with men
- Acknowledge the sensitivity of GBV. Also acknowledge that men can and are sometimes the victims of GBV, including in Kenya, but that this work was more focused on men as perpetrators of violence (given research knowledge on the topic)
- Confirmation of anonymity, voluntary participation and input to the discussions as being optional.
- Seek verbal informed consent.

*Topic 1: Explore why individuals felt men perpetrated GBV, particularly intimate partner violence:*

- How do men view GBV?
- How would you know if a man was perpetrating GBV?

*Topic 2: Mental or emotional problems facing men in Waithaka/Muitini:*

- What mental or emotional problems are affecting men in Waithaka/Muitini?
- How and where might men seek help for mental or emotional problems?

*Open discussion:*

- What else should we know about in relation to men in these communities and/or GBV?
